# Stock-specific chemical brood signals are induced by *Varroa* and Deformed Wing Virus, and elicit hygienic response in the honey bee

**DOI:** 10.1038/s41598-019-45008-2

**Published:** 2019-06-19

**Authors:** K. Wagoner, M. Spivak, A. Hefetz, T. Reams, O. Rueppell

**Affiliations:** 1 Biology Department, University of North Carolina at Greensboro, Greensboro, USA; 2 Department of Entomology, University of Minnesota, Minneapolis, USA; 3 George S. Wise Faculty of Life Science, Tel Aviv University, Tel Aviv, Israel; 4 Department of Entomology, Texas A&M University, College Station, USA

**Keywords:** Chemical ecology, Behavioural ecology

## Abstract

The health of the honey bee *Apis mellifera* is challenged by the ectoparasitic mite *Varroa destructor*, and the numerous harmful pathogens it vectors. Existing pesticide-based *Varroa* controls are not sustainable. In contrast, one promising approach for improved honey bee health is the breeding of hygienic bees, capable of detecting and removing brood that is parasitized or diseased. In three experiments we find evidence to support the hypothesis that stock-specific chemical brood signals are induced by *Varroa* and Deformed Wing Virus, and elicit hygienic response in the honey bee. By collecting, analyzing, and running bioassays involving mite-infested and control brood extracts from three honey bee breeding stocks we: 1) found evidence that a transferrable chemical signal for hygienic behavior is present in *Varroa*-infested brood extracts, 2) identified ten stock-specific hydrocarbons as candidates of hygienic signaling, and 3) found that two of these hydrocarbons linked to *Varroa* and DWV were also elevated in brood targeted for hygienic behavior. These findings expand our understanding of honey bee chemical communication, and facilitate the development of improved hygienic selection tools to breed honey bees with greater resistance to *Varroa* and associated pathogens.

## Introduction

While demand for crop pollinators increases^1,2^, the health of the honey bee *(Apis mellifera)* is declining worldwide^3^. Recent declines in honey bee health are attributed to a number of factors, including the introduction and spread of parasites and their associated pathogens^4,5^. The obligate, ectoparasitic honey bee mite *Varroa destructor* (*Varroa*) is the most important biological threat to honey bee health today^6,7^. *Varroa* evolved as a parasite of the Asian honey bee *Apis cerana* and has expanded to the European honey bee *Apis mellifera*, resulting in a near global distribution^8^.

In order to reproduce, foundress *Varroa* mites enter the cells of uncapped 5^th^ instar larvae, and conceal themselves at the base of the cell. After the cell has been capped by nurse bees, the mite emerges and begins to feed on the larva^9^. The mite lays her first egg approximately 70 hours after capping of the honey bee cell. The first egg is haploid and develops into a male. Diploid eggs follow at approximately 30-hour intervals, and develop into females^7^. Subsequent sib-mating leads to development of fertilized female *Varroa* offspring in the parasitized brood cell^7^.

*Varroa* feeding is harmful to honey bee health, and can lead to decreases in body weight and protein levels^10–12^. *Varroa* mites also vector honey bee pathogens^13–16^, and have been associated with the amplification and increased susceptibility of honey bees to viruses^13,16–18^. The most common among them, Deformed Wing Virus (DWV), is a positive, single stranded RNA virus^19^ associated with deformed wings, a shortened abdomen, reduced weight, discoloration, and premature death^11,20,21^. In the absence of *Varroa*, DWV is typically asymptomatic^13,18,21,22^, however DWV that is associated with *Varroa* is a significant contributor to decline in honey bee health^16^.

As eusocial insects, honey bees complement individual immunity with mechanisms of social immunity for defense against parasites and pathogens. For example, many brood diseases can elicit the age-specific antiseptic activity known as *hygienic behavior*. Hygienic behavior is the detection, uncapping, and removal of diseased brood (larvae or pupae) from the hive^23–25^, and is most commonly observed in worker bees aged 15 to 20 days^26^. Breeding programs have enhanced *Varroa* resistance in some honey bee stocks through selection for hygienic behavior. Two such hygienic stocks are the Minnesota Hygienic bees (HYG), selected from high-performing unselected (UNS) colonies based on the removal of freeze-killed brood^27^, and the *Varroa* Sensitive Hygienic bees (VSH), selected from high-performing UNS colonies based on apparent suppression of mite reproduction^28^.

While selection based on the suppression of mite-reproduction has been largely successful at reducing mite loads of VSH colonies, the selection methods are quite labor intensive. In contrast, assays based on the removal of freeze killed brood are much easier to perform, but do not achieve the same level of mite resistance; miticides are still needed to control severe mite infestations in HYG colonies^29,30^. However miticides are not a sustainable solution, as they are harmful to honey bee health^31–35^, and mite populations rapidly evolve resistance to the chemicals^7,36^. One sustainable solution for enhanced *Varroa* control would be to improve selective breeding methods^37–40^ through the development of a simpler method to select for the same level of *Varroa* control as is currently found in VSH colonies.

The role of heightened olfactory sensitivity of adult bees in enhanced removal rates by hygienic colonies has been well established through behavioral, neuronal, proteomic, and transcriptomic studies. Specifically, nurse bees from hygienic colonies and/or performing hygienic behavior have been associated with increased sensitivity to diseased brood odor^41,42^, greater immunoreactivity to the neuromodulator octopamine^26,43^, proteomic changes in mushroom bodies^44^, up-regulation of some odorant binding proteins^44^, and overrepresentation of some genes related to cell signal transduction and olfactory perception^45,46^. However to date, little is known about the signals that guide nurse bees to perform hygienic behavior.

The stimulus for hygienic behavior may involve chemicals from the brood cuticle^47–50^. In *Varroa*-infested cells, it is apparent that foundress mites and their offspring play a role in eliciting hygienic behavior^51,52^. However mites are known to mimic cuticular chemical profiles of brood^53–55^, which likely makes them difficult to detect directly. Aumeier *et al*.^47^ found no evidence that hygienic behavior resulted from odor or movement of *Varroa*, suggesting instead that the stimulus for hygienic behavior might originate from the brood. Furthermore, hygienic behavior occurs in response to a variety of diseases^23,50,56,57^, several of which are unrelated to *Varroa*. Additionally, cross-fostering studies indicate that in hygienic behavior, the brood stock can be more influential than that of the nurses^58^. β-ocimene and oleic acid are released from liquid nitrogen-frozen honey bee brood and can be used to elicit hygienic behavior^59^. However, β-ocimene is a general attractant that is also produced by healthy larvae to solicit feeding^60^ and oleic acid is a general insect necromone^61,62^. Therefore, these substances may not represent the potentially less conspicuous stress signals of living brood in response to *Varroa* and/or virus-infection.

The cuticles of honey bees and other Hymenoptera are typically dominated by hydrocarbons^63–65^, which are transferred via the hemolymph from the oenocytes to the insect cuticle^66^, where they function both to prevent desiccation and facilitate communication^63,67–69^. When used for communication, CHC type and quantity can be discriminated with varying degrees of sensitivity for inter- and intra-colonial recognition tasks^70^. For example, larger or more easily detected differences in CHC profiles may be required to discriminate nestmates from intruders^71–73^, while more discrete differences, such as differences in the relative quantities of single compounds, may be sufficient to discriminate intra-colonial age and caste designations^70,72^. Baracchi *et al*.^74^ found evidence of removal of DWV-infected adult bees from the colony, and linked DWV infection to changes in the CHCs of adult bees, specifically, an increase in higher molecular weight CHCs. In honey bee brood, *Varroa* infestation and DWV levels of infesting *Varroa* have been linked to quantitative CHC changes in the brood. Salvy *et al*.^49^ found that *Varroa*-parasitized larvae had higher quantities of 10-C33:1 (tritriacontene) and 10 + 9-C31:1 (hentriacontene). Similarly, Nazzi *et al*.^75^ reported an increase in the short chain alkenes C15:1 (pentadecene) and C17:1 (heptadecene) in response to *Varroa* infestation, and showed that at least one isomer of pentadecene (6-C15:1) could elicit hygienic removal when applied to cells. Schöning *et al*.^50^ found that brood associated with mites with high-DWV titers were more likely to be removed, and could be differentiated from non-infested brood by headspace monoterpene hydrocarbons. These findings support the notion that stressor-induced differences in cuticular chemistry lead to damage-dependent removal of honey bee brood. However, more information is needed to identify the natural chemosensory signals that elicit hygienic behavior.

Here, we tested the hypothesis that stock-specific chemical brood signals are induced by *Varroa* and Deformed Wing Virus, and elicit hygienic response in the honey bee. Specifically, we predicted that 1) hygienic uncapping can be induced using *Varroa*-infested honey bee brood extracts, 2) *Varroa* and DWV quantitatively affect brood CHCs, 3) effects of *Varroa* and DWV on brood CHCs vary by honey bee stock, and 4) CHCs elevated in the presence of *Varroa* and/or DWV are particularly elevated in brood targeted for hygienic uncapping. We tested this hypothesis by performing a set of three experiments, which provided support for our predictions, and thus identify specific honey bee brood CHCs as the likely signals eliciting hygienic removal.

## Results

### Experiment 1: Effects of Varroa-infested honey bee brood extracts on hygienic uncapping behavior

**Figure 1:**
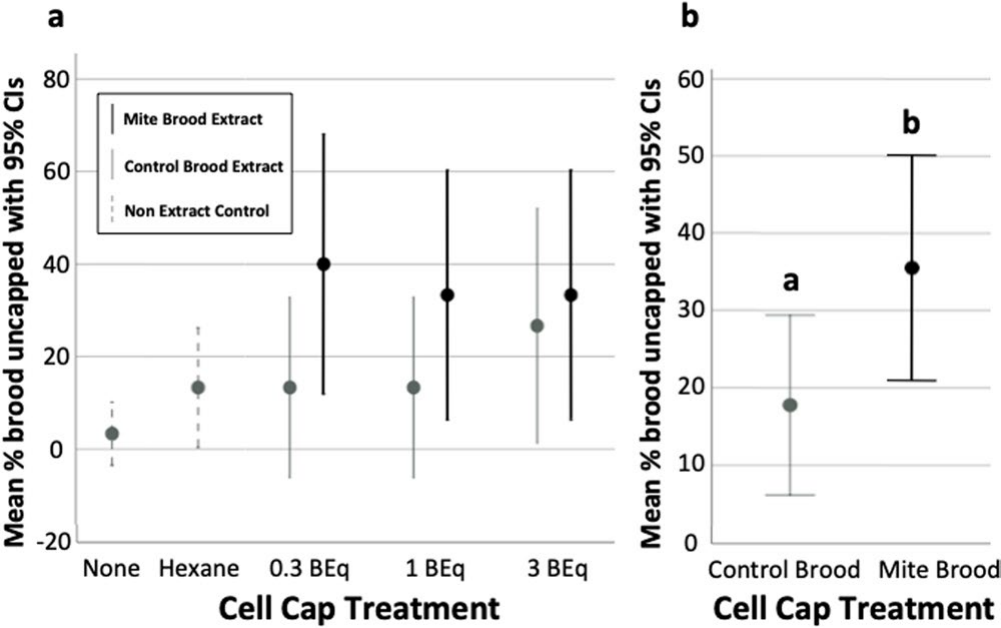
Evidence for a chemical stimulus for hygienic behavior in a VSH colony, from Experiment 1. For each mean, 95% CI intervals are provided. Different letters indicate a significant difference in mean percent brood uncapped, from Chi-square analyses. (**a**) Mean percent of brood cells uncapped 8 hours after applying no treatment, hexane (solvent) treatment, or brood extract treatments from mite-infested or control brood. Extracts from mite-infested brood caused a higher proportion of uncapping than either of the control treatments, but not in a dose-dependent manner. Cell sample sizes: n = 30 for negative control and hexane control, n = 15 for each of three concentrations of the control extract and mite-infested extract treatments. There were no significant differences in pairwise comparisons between mite-infested and non-infested treatments for each concentration (**b**) Over all concentrations, significantly more cells treated with mite-infested brood extract were uncapped than cells treated with control extract (p = 0.029).

In Experiment 1 we investigated whether transferring extracts of *Varroa*-infested VSH brood onto brood cell caps elicited hygienic behavior in a VSH colony, addressing our first prediction. The frequency of uncapping was consistently higher in cells treated with any of the three concentrations of mite-infested brood extract than in untreated cells, hexane-treated cells, and cells treated with the respective concentrations of control brood extract. No dose-dependency was observed, and none of the pairwise comparisons among treatments within each concentration was significant (Fig. 1a). Overall, cells treated with mite-infested brood extract were uncapped significantly more often than cells treated with control extract (Χ^2^ = 3.64, d.f. = 1, p = 0.029, n = 90; Fig. 1b).

### Experiment 2: Effects of Varroa, DWV, and Brood Stock on Honey Bee Brood CHCs

**Figure 2:**
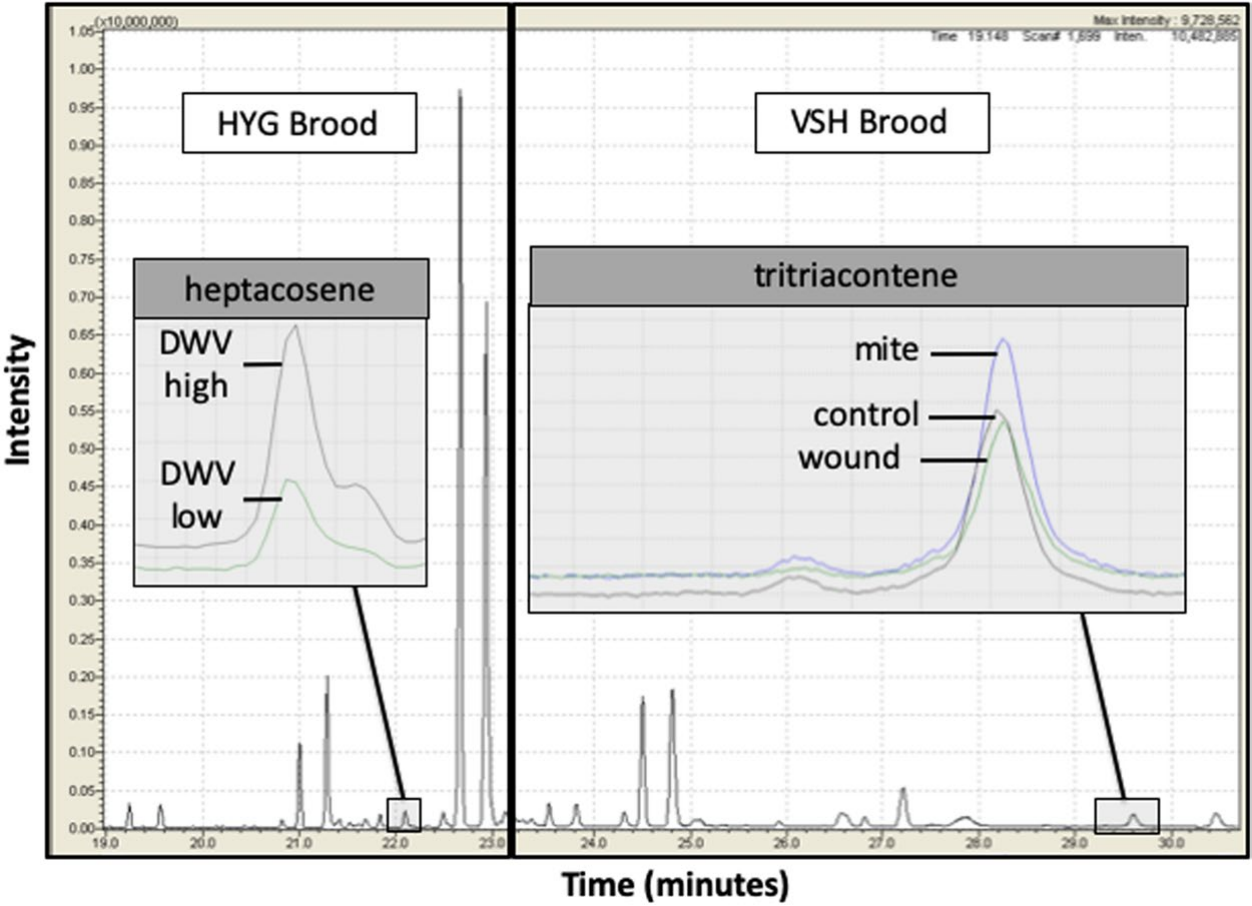
Example of (non-normalized) chromatogram illustrating the increase in heptacosene in HYG brood with high DWV, and the increase in tritriacontene in *Varroa*-infested VSH brood.

**Figure 3:**
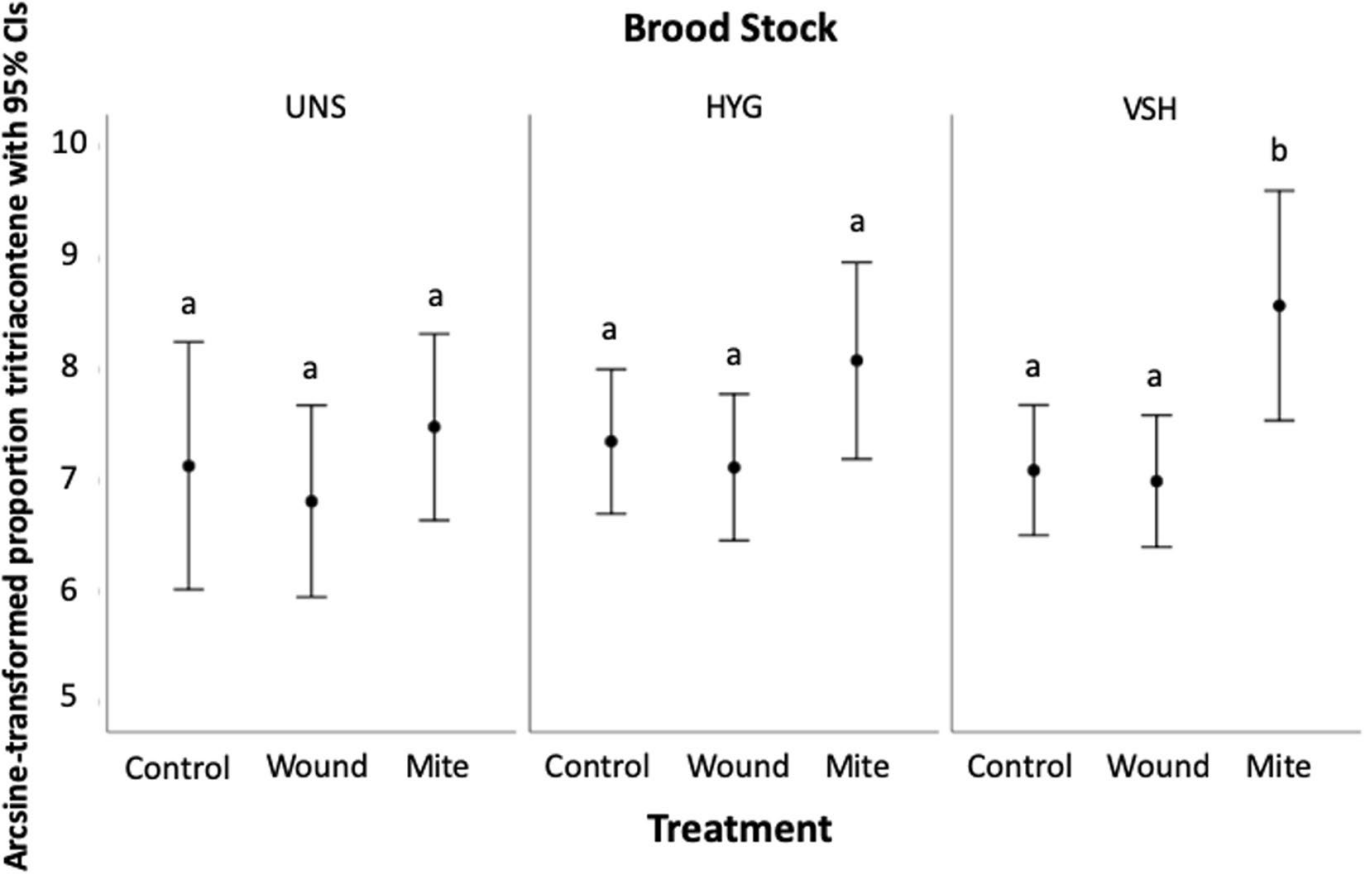
Mean tritriacontene proportion in control, wounded, and mite-infested brood for UNS, HYG, and VSH Brood Stocks, from Experiment 2. For each mean, 95% CI intervals are provided. Mite, wound, and control sample sizes were 67, 31, and 30 for UNS brood, 54, 55, and 54 for HYG brood, and 56, 53, and 51 for VSH brood. Different letters indicate a significant difference in mean tritriacontene proportion (relative to the total chemical quantity) between treatment groups within each brood stock, from an ANOVA with Bonferroni correction (p < 0.0167). Tritriacontene is shown as an example because it was the only chemical elevated in response to both stressors and in all three brood stocks.

In Experiment 2 we analyzed the quantitative effects of *Varroa* and Deformed Wing Virus (DWV) on the cuticular hydrocarbon (CHC) profiles of brood from VSH, HYG, and UNS breeding stocks, addressing our second and third predictions. A total of 33 chemicals from the cuticles of honey bee brood were characterized and quantified in this experiment (Table 1, Supp. Fig. S1). Of these, 12 were alkanes, 7 were alkenes, 13 were methylalkanes, and 1 was unidentified. Although a few variables violated normality, parametric tests were used throughout for consistency to assess complete statistical models, including interaction terms and multivariate effects. There was a significant effect of treatment on brood cuticular profiles (λ_Wilks_ = 0.776, F_(66,754)_ = 1.626, p = 0.002). Of the 33 chemicals analyzed, 2 were significant for treatment effects (Table 1, Supp. Table S1). However, after Bonferroni correction in pairwise comparisons, treatment differences were only significant for tritriacontene, where the proportion of tritriacontene was significantly higher in mite-infested brood than in control (p = 0.007) or wounded (p = 0.001) brood (Fig. 2), with no difference between wounded and control brood (p = 1.000). When we analyzed each stock separately, treatment had a significant effect on tritriacontene proportion in VSH brood (F_(2,156)_ = 5.230, p = 0.006) but not in HYG (F_(2,160)_ = 1.861, p = 0.159) or UNS (F_(2,87)_ = 0.858, p = 0.427) brood. Tritriacontene was significantly higher in mite-infested VSH brood than wounded (p = 0.011) or control (p = 0.025) brood (Fig. 3). No significant difference in tritriacontene proportion was found between wounded and control VSH brood (p = 1.000), or between treatments in HYG or UNS brood (Fig. 3). When the wound treatment was removed results were similar; mite treatment had a significant effect on tritriacontene proportion in VSH brood (F_(1,104)_ = 5.983, p = 0.016) but not in HYG (F_(1,106)_ = 1.783, p = 0.185) or UNS (F_(1,57)_ = 1.335, p = 0.253) brood. The direction of differences in compound quantities between mite-infested and negative control brood are reported (Table 1, Supp. Table S1).

**Table 1:** Summary of CHC analyses from Experiments 2 and 3.

	Experiment 2 *Mite*			Experiment 2 *DWV*			Experiment 3 *Hygiene*
PEAK	UNS	HYG	VSH	UNS	HYG	VSH	VSH
unidentified*	+	+	+	+	1/2+	1/2+	−
nonadecane	−	−	−	−	+	+	−
heneicosane	−	+	−	−	+	−	−
tricosane	−	+	−	+	+	+	+
9- + 11-methyltricosane	−	+	−	2/2+	1/2+	−	−
4-methyltetracosane	−	+	−	2/2+	+	+	−
pentacosene*	−	+	+	2/2+	2/2+	+	+
pentacosane*	+	−	−	1/2+	+	+	+
11- + 13-methylpentacosane	−	−	+	2/2+	2/2+	−	+
hexacosane*	−	−	−	2/2+	+	+	+
12- + 14-methylhexacosane	−	−	+	2/2+	2/2+	−	+
heptacosene*	−	−	−	2/2+	2/2+	−	+
heptacosane*	+	+	−	−	2/2−	−	−
11- + 13-methylheptacosane	−	−	−	+	−	−	+
5-methylheptacosane^#^	−	−	−	−	+	1/2−	+
11,15-dimethylheptacosane	−	−	−	+	+	−	+
7,x-dimethylheptacosane	−	−	−	−	−	−	+
5,x-dimethylheptacosane	−	+	−	−	−	−	+
octacosane	−	+	−	+	−	−	+
nonacosene	−	−	−	+	+	−	+
nonacosane*	+	−	−	−	2/2−	−	−
11- + 13- + 15-methylnonacosane	+	−	−	2/2−	−	−	+
11,17-dimethylnonacosane	−	−	−	+	−	+	−
triacontene	−	−	−	+	−	+	−
triacontane	−	−	−	−	+	+	−
hentriacontene	−	−	+	2/2+	+	−	3/3+
hentriacontane	+	+	+	−	−	−	+
11- + 13 - + 15-methylhentriacontane	+	−	−	−	−	+	+
13,17-dimethylhentriacontane	−	−	−	−	−	+	−
dotriacontene	+	+	+	+	−	−	−
methyldotriacontane	+	−	−	−	−	+	+
tritriacontene^#^*	+	+	3/3+	2/2+	2/2+	−	3/3+
tritriacontane	−	−	−	2/2−	+	+	+

Significant treatment and DWV effects over all brood stocks are represented by number signs and asterisks, respectively. Plus and minus signs are used to indicate the direction of the change in chemical proportions associated with treatment (mite vs. control), DWV quantity, or hygienic behavior (uncapped mite vs. capped mite). For chemicals associated with statistically significant effects in individual brood stocks, numbers in the cells represent the number of years with the same trend direction over the number of years the experiment was performed.

**Figure 4:**
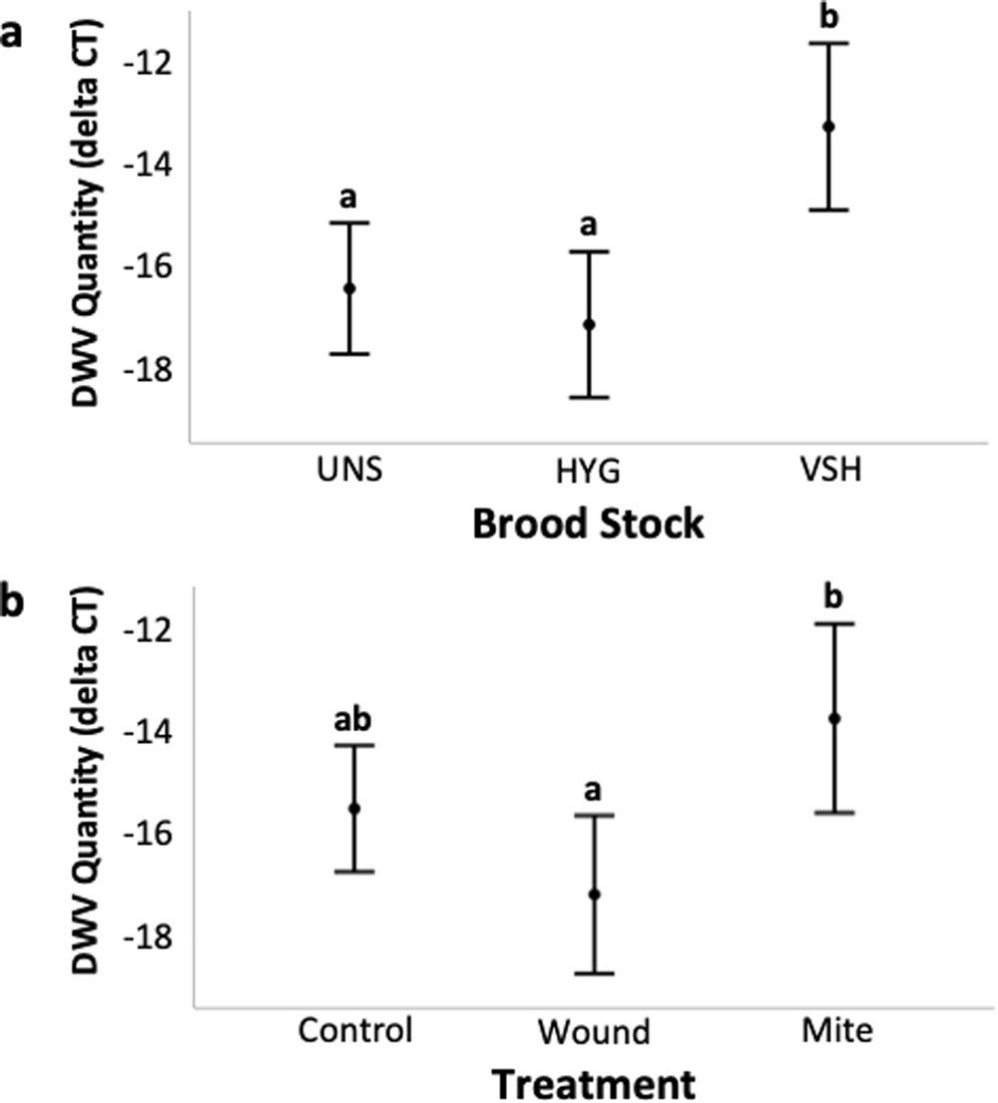
Effects of Brood Stock and Treatment on DWV quantity, from Experiment 2. For each mean, 95% CI intervals are provided. Different letters indicate a significant effect of stock or treatment on mean DWV quantity, from a two-way ANOVA with Bonferroni corrected post-hoc analysis. (**a**) Mean DWV quantity of UNS, HYG, and VSH brood (n = 91, 126, and 124, respectively). (**b**) Mean DWV quantity of Control, Wound and Mite treatments (n = 140, 102, 99 respectively).

DWV quantity differed significantly among honey bee stocks (F_(2,332)_ = 8.873, p < 0.001), with significantly higher DWV in VSH colonies than in HYG (p < 0.001) or UNS (p = 0.012) colonies (Fig. 4a). There was no significant difference between DWV levels in HYG and UNS colonies (p = 1.000). There was suggestive evidence that treatment affected DWV quantity (F_(2,332)_ = 2.972, p = 0.053), where DWV level was higher in mite-infested than in wounded (p = 0.007) brood (Fig. 4b). No difference in DWV level was found between mite-infested and control (p = 0.273), or wounded and control (p = 0.312) brood.

**Figure 5:**
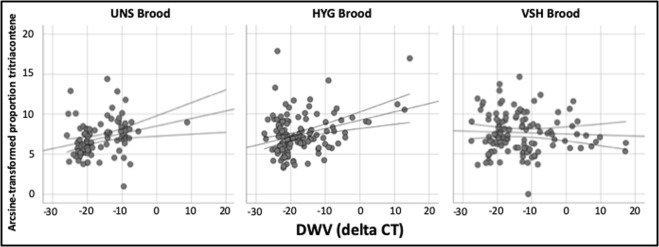
DWV vs. tritriacontene proportion in UNS, HYG, and VSH brood, from Experiment 2. Circles represent individual brood. Lines of best fit with 95% CI intervals of the mean are provided. Significant correlations were observed for CON (Pearson’s R = 0.263, n = 91, p = 0.012) and HYG (R = 0.322, n = 126, p < 0.001) but not VSH (R = −0.040, n = 124, p = 0.659) brood. Tritriacontene is shown as an example because it was the only chemical elevated in response to stressors in all three brood stocks.

DWV quantities were significantly correlated with 8 chemicals across all samples (Table 1, Supp. Table S1). Of these 8 chemicals, 6 (2 alkanes, 3 alkenes, and 1 unidentified) were positively correlated with DWV, including heptacosene and tritriacontene (Fig. 2). When brood stocks were analyzed separately, significant DWV effects were identified for 12, 9, and 2 chemicals in UNS, HYG, and VSH brood, respectively (Table 1, Supp. Table S1). For tritriacontene, the only chemical elevated in response to stressors in all three brood stocks, significant correlations with DWV quantity were observed for CON (Pearson’s R = 0.263, n = 91, p = 0.012) and HYG (R = 0.322, n = 126, p < 0.001) but not VSH (R = −0.040, n = 124, p = 0.659) brood (Fig. 5). The effects of DWV on UNS and HYG brood chemicals were more similar to each other than to those on VSH brood chemicals (Table 1).

Even with limited experimental manipulation (negative control treatment) the CHC profile of brood stocks differed significantly (λ_Wilks_ = 0.304, F_(33,66)_ = 2.540, p < 0.001). One-way ANOVAs indicated that of the 33 chemicals analyzed, 6 were significant for stock effects. However, after Bonferroni correction in pairwise comparisons, stock differences were only significant for 5 chemicals: pentacosane, hexacosane, 11- + 13-methylheptacosane, 5,x-dimethylheptacosane, and dotriacontene. Only 2 of these - pentacosane and hexacosane - were also associated with treatment effects. In both cases, differences were only significant between UNS and VSH colonies (p = 0.046 and p = 0.027 for pentacosane and hexacosane, respectively).

### Experiment 3: Hygiene-associated Brood CHCs

**Figure 6:**
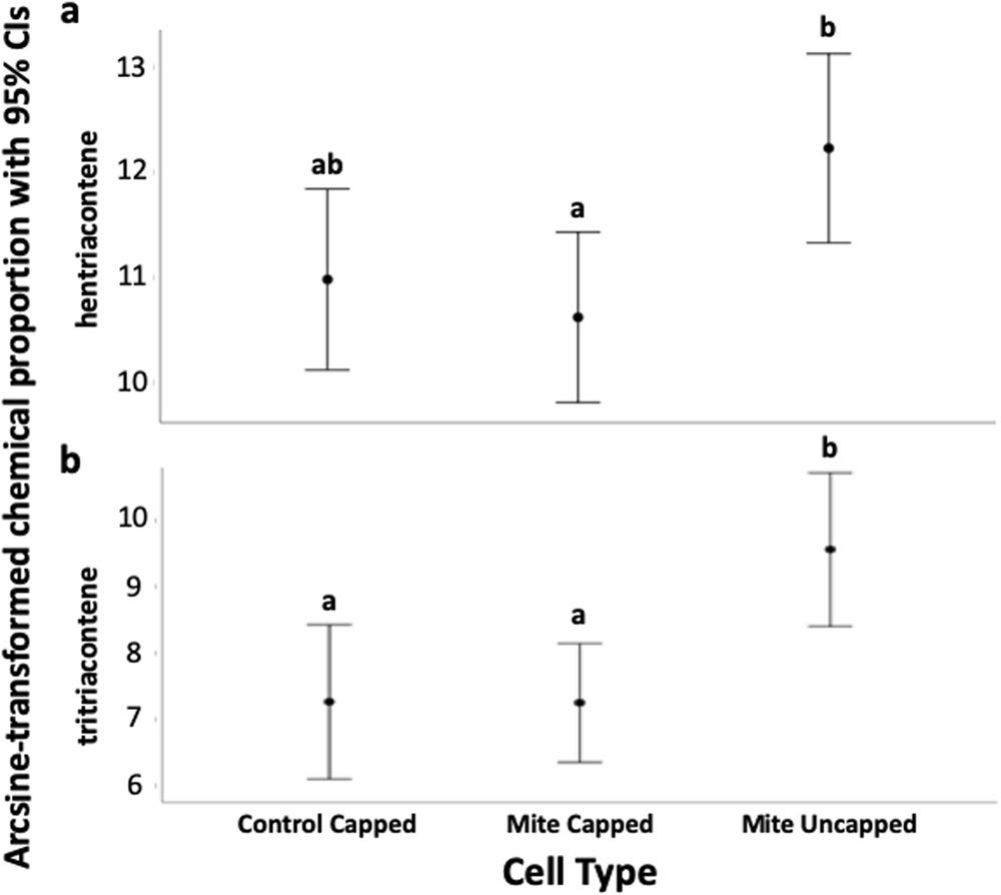
Evidence of a link between chemical proportion and hygienic behavior, from Experiment 3. Sample size was 30 brood per Cell Type. For each mean, 95% CI intervals are provided. Different letters indicate significant differences in mean chemical proportion between cell types, from an ANOVA with Bonferroni correction. (**a**) Mean hentriacontene proportion (relative to the total chemical quantity) of VSH brood from control capped, mite capped, and mite uncapped cells. (**b**) Mean tritriacontene proportion (relative to the total chemical quantity) of VSH Brood from control capped, mite capped, and mite uncapped cells.

In Experiment 3 we determined which CHCs were elevated in VSH brood targeted by nurse bees for hygienic behavior, addressing our fourth prediction. Brood cuticular profiles differed significantly by cell type (λ_Wilks_ = 0.273, F_(66,110)_ = 1.526, p = 0.025). When all three cells types (uncapped infested, capped infested, and capped non-infested) were included, one-way ANOVAs indicated that mean proportion of hentriacontene and tritriacontene differed significantly by cell type (F_(2,87)_ = 4.05, p = 0.021 and F_(2,87)_ = 6.35, p = 0.003, respectively). In pairwise comparisons, the mean proportion of hentriacontene was significantly higher in uncapped infested brood than in capped infested brood (p = 0.024), but there were no significant differences in the mean proportion of hentriacontene between uncapped infested and capped non-infested brood (p = 0.115), or capped infested and capped non-infested brood (p = 1.000) after Bonferroni correction (Fig. 6a). In pairwise comparisons, the mean proportion of tritriacontene was significantly higher in uncapped infested brood than in both capped infested brood (p = 0.008) and capped non-infested brood (p = 0.008), but no significant difference in the mean proportion of tritriacontene was found between capped infested and non-infested brood (p = 1.000; Fig. 6b). There were no other significant effects of the three experimental cell types on CHC proportions. When capped control cells were excluded from the analysis, mean proportion of hentriacontene and tritriacontene differed significantly by cell type (F_(1,58)_ = 7.375, p = 0.009 and F_(2,58)_ = 10.432, p = 0.002, respectively), and there were no other significant differences in CHC proportions (Table 1, Supp. Table S1). While there was suggestive evidence that mite-infested cells had higher DWV quantities than non-infested cells (F_(1,79)_ = 3.228, p = 0.076), no significant effect of cell type on DWV quantity (F_(1,78)_ = 1.594, p = 0.210) was observed (Supp. Fig. S2).

## Discussion

Chemical communication is essential for the coordination of complex behaviors in social insects. Here we provide evidence that a signal for honey bee hygienic behavior involves quantitative changes in brood cuticular chemistry, and identify several specific compounds with higher proportions on the cuticles of *Varroa* parasitized and/or DWV-stressed brood. Though the majority of the chemicals that increased significantly in response to stress were conserved between at least two honey bee stocks, our results indicate that the stressor that induces a chemical response may differ according to stock selection methods. Additionally, we show that two chemicals elevated in response to stressors were also elevated in brood targeted for hygienic behavior. Previous studies have linked variation in brood chemicals to mite infestation^49,63^, DWV infection^50^, and hygienic behavior^59,75^. Had the chemicals detected here been derived from mites and their offspring, we would have expected to see a *Varroa* effect in all stocks, and would not have expected a similar chemical response to DWV. To our knowledge, this is the first study to link the same chemicals to multiple honey bee brood stressors and to hygienic behavior, or to show that honey bee stock can affect brood chemical stress signals. Our results indicate that variation in hygienic removal may be influenced by differences in the ability of brood to signal stress via quantitative changes in CHC production. Identification of the natural chemical signal eliciting honey bee hygienic behavior may be useful in the development of improved hygienic selection tools, and thus to the improvement of honey bee health through enhanced removal of parasitized and diseased brood.

Our hypothesis that stock-specific chemical brood signals are induced by *Varroa* and Deformed Wing Virus, and elicit hygienic response in the honey bee is supported by results from all three experiments performed. Our first prediction was that hygienic uncapping could be induced by treating brood cell caps with extracts of *Varroa*-infested honey bee brood. Correspondingly, in Experiment 1 we observed higher uncapping rates of cells treated with extract from *Varroa*-infested brood than of those treated with extract from control brood. While this trend was consistent for all extract concentrations tested, the difference in uncapping was only statistically significant when results from all concentrations were combined. Uncapping frequencies of cells treated with the two lower control brood extract concentrations were the same as that of hexane-treated cells. The higher uncapping frequency of cells treated with the highest concentration of control brood extract may have been a general response of the nurse bees to the abnormally high amount of brood chemicals on the cell cap. Despite our relatively small sample size, our data support the growing body of evidence suggesting that the signal eliciting hygienic response is contained within non-polar extracts of brood cuticles^47–49^.

In Experiment 2, we examined the effects of *Varroa*-infestation and DWV quantity on the CHC profiles of brood from three different stocks to test our prediction that *Varroa* and DWV quantitatively affect brood CHCs. We identified ten chemicals whose proportion increased significantly in response to common honey bee stressors in at least one honey bee stock; two alkanes (pentacosane and hexacosane), four methylalkanes (4-methyltetracosane, 9- + 11-methyltricosane, 11- + 13-methylpentacosane, 12- + 14-methylhexacosane) and four monounsaturated alkenes (pentacosene, heptacosene, hentriacontene, and tritriacontene). Saturated hydrocarbons may have evolved to provide desiccation resistance^76^, but methyl-branched and unsaturated hydrocarbons likely serve alternate functions on insect cuticles, such as intra- and inter-specific communication^70^. The role of methyl-branched and unsaturated hydrocarbons in communication is consistent with our evidence of their contribution to social immune signaling. Our observations of stressor-associated increases in pentacosene, heptacosene, hentriacontene, and tritriacontene are also consistent with reports associating alkenes with *Varroa* infestation^75^, including previous associations of *Varroa*-infested brood with hentriacontene and tritriacontene^49^. Monomethylalkanes and dimethylalkanes have also previously been associated with *Varroa* and *Varroa* parasitization in honey bee brood^77^. The branched fatty-acid precursors of branched hydrocarbons have strong antimicrobial properties^78^, which may explain their positive association with DWV levels. However such a relationship is merely speculative, as the contribution of fatty acids to honey bee immune response was not tested here.

Experiment 2 also addressed our third prediction that, due to artificial selection, effects of *Varroa* and DWV on brood CHCs vary by honey bee stock. In UNS and HYG brood, no significant response to *Varroa* infestation was observed. However in UNS brood, DWV was positively associated with increases in 10 CHCs. In HYG brood, 6 of these CHCs were also positively associated with DWV. In contrast, VSH brood showed an increase in 1 CHC in response to *Varroa* infestation, but no significant response to DWV was observed in any of the CHCs we identified. These stock-specific variations in CHC response are likely a result of stock selection methods, where selection for VSH seems to have had a significant positive effect on brood signaling related to *Varroa* infestation, and a significant negative affect on brood signaling related to DWV infection. This idea is supported by evidence of a chemical response to *Varroa* by VSH brood, which is selected for at the colony level using a method directly linked to *Varroa*^79^. The lack of VSH brood response to DWV may have been related to variation in the DWV strain(s) present, although this was not tested, as the primers for DWV strains A, B, and C^80^ were not available at the time of our analyses. Though it is not tested here, positive selection for non *Varroa*-associated DWV strains could reduce DWV virulence^22,81^ through virus-virus competition^82^ in VSH colonies, a theory consistent with our findings that VSH colonies 1) did not signal DWV infection 2) had the highest DWV levels of any colony type, and 3) had significantly higher DWV levels than HYG brood in control and wound but not mite treatments. Alternatively, VSH bees could be more susceptible to DWV than other stocks. Future studies are needed to explore the interactions between DWV strains, brood signaling, and brood stock.

The similarity in the response of HYG and UNS brood to DWV (but not *Varroa* infestation) indicates that hygienic response selected for in HYG bees is primarily dependent on adult chemosensory capability (colonies exhibiting greater response to the odors of dead brood), and not brood signaling ability. This is consistent with evidence of enhanced olfactory sensitivity in adults performing hygienic tasks^26^, as well as with reports that there are more hygienic task performers in colonies selected for hygiene based on killed brood^46^. Furthermore, the reduction from 10 chemicals signaling DWV infection in UNS brood to 6 chemicals signaling DWV infection in HYG brood may indicate that selection using the freeze-killed brood-assay can unintentionally lead to the selection of colonies containing brood with a *reduced* ability to signal stress, through coevolution between signal production and detection^58^. This coevolution would imply that colonies have either a combination of weak signalers and strong detectors or a combination of strong signalers and weak detectors. By selecting colonies based on adult performance, the freeze-killed brood assay may have selected for the former. Since dead brood likely emit stronger signals than parasitized brood^41,83,84^, this coupling of high adult olfactory ability with low brood signaling ability is consistent with the reported disparity between the removal of freeze-killed brood and *Varroa*-infested brood in HYG colonies^85^. However, HYG bees are also able to detect and remove brood in early stages of American Foul Brood^23^ and Chalkbrood^56^ infections, in contrast to the hypothesis of hygienic sender/receiver coevolution. Additional research is needed to understand how different selection methods may have affected signal production/detection coevolution, and if and to what extent chemical brood signaling is involved in the detection of additional honey bee pathogens.

Our fourth prediction was that CHCs that were elevated in the presence of *Varroa* and/or DWV would be particularly elevated in brood targeted for hygienic uncapping. Experiment 3 results revealed significantly increased proportions of hentriacontene and tritriacontene in mite-infested VSH brood targeted for hygienic removal compared to capped mite-infested and non-infested VSH brood. Since the presence of DWV in uncapped mite-infested cells was not greater than that of capped mite-infested cells, there is no indication that DWV affects the selection of brood for uncapping in VSH colonies, consistent with results from Experiment 2 and previously published findings^52^. Hentriacontene and tritriacontene were elevated in the presence of *Varroa* and DWV in Experiment 2, and linked to VSH uncapping behavior in Experiment 3. Whether hentriacontene and tritriacontene are associated with hygienic behavior in non-VSH colonies remains to be tested.

Tritriacontene has also been associated with the unpacking of ant pupae^86^, which is a behavior comparable to hygiene in honey bees. Interestingly, many of the compounds associated with stressors and hygienic (or unpacking) behaviors in this and previous studies are hydrocarbons with an odd-numbered chain length, and a single double bond located on an even-numbered carbon^49,75,86^. While signaling ability and sensitivity to various stressors may differ between honey bee colonies and stocks, similarities in the chemical structure of stress signals across stocks is consistent with evidence of a unified response to multiple pathogens in diseased honey bee nurses^87^. This suggests that specific CHC stress signals may be conserved across multiple social insect species, although more studies are needed to confirm or refute this hypothesis.

One important aspect of hygienic communication that remains unresolved is the mechanism by which a signal emitted from within a capped brood cell reaches a nurse bee on the other side of the cell cap. The porous nature of honey bee cell caps likely enables air flow between the inside and outside of the brood cell^88^, which may facilitate communication between capped brood and patrolling nurses. However the low volatility of many of the chemicals associated with hygienic behavior may mean that the lipophilic compounds bind to and move through the wax cap without volatilizing. Martin *et al*.^77^ reference unpublished results indicating that brood cuticles and associated brood cell caps have similar chemical compositions, and suggest that even if chemicals are minimally volatile, patrolling nurses may be able to detect subtle differences in cell-specific chemical compositions. Since hygienic removal is fatal to the targeted brood, it makes evolutionary sense for hygienic signals to be highly localized (and thus less volatile), reducing the frequency of accidental removal of healthy brood. However additional volatile signals may be useful for attracting nurses that exhibit hygienic behavior to the general area where a pest or pathogen is present. Such chemical synergy through volatility mechanics has been suggested previously^59^, and is a likely explanation of the contribution of the broad range in carbon chain lengths of hygiene-associated CHCs.

In sum, results from all experiments support the notion that stressors affect honey bee brood CHCs, and that changes in brood CHCs in response to various stressors contribute to eliciting hygienic behavior in a stock-dependent manner. Thus, different selection practices for disease and pest control may have altered brood signals in addition to signal responsiveness of nurses^26,45^, indicating that strong artificial selection for increased hygienic behavior in honey bees can lead to co-evolutionary responses between sender and receiver. Most social traits, such as hygienic behavior, rely on communication. Therefore, such correlated micro-evolutionary changes could be common in social insects. Furthermore, findings from the experiments reported here may be useful in the development of novel tools to treat and improve colony resistance to honey bee parasites and diseases. For example, a chemical stimulus for uncapping without removal could reduce *Varroa* fitness through disruption of mite reproductive success^89,90^ and increased *Varroa* removal, since *Varroa* are easier to detect by a greater number of nurses in uncapped cells^41^. Furthermore, the complexity and generality of current selection methods^39,83^ may be improved upon through the development of new selection assays, which utilize uncapping and/or removal of brood from chemically treated cells as the primary selection criteria for hygienic colonies from which to breed. Since removal rates are lower for mite-infested brood than for freeze-killed brood^84,91^, it follows that the olfactory signal eliciting hygienic behavior is lower in live brood than in dead brood^83^. This is further supported by the higher volatility (as determined by vapor pressure) of β-ocimene and oleic acid released by dead brood^59^ than of hentriacontene and tritriacontene released by stressed, living brood. Thus, theoretically, colonies that uncap and/or remove more cells resembling those with stressed, live brood would contain bees with higher sensitivity to diseased brood odor, and thus be more effective at detecting and targeting low intensity stress signals of *Varroa*-infested and DWV-infected brood.

Development of techniques that enhance the control of honey bee parasites and diseases should improve honey bee health, increase colony population sizes, and facilitate overwintering success^92,93^. Improved honey bee health and survival have major economic implications, given the critical role that honey bees play in the pollination and yield of dozens of important crops around the world. In addition to providing the foundation needed for the development of tools that improve control of honey bee parasites and diseases, this study improves our understanding of honey bee communication, and may provide useful insights regarding active compounds, olfactory sensitivity, and the fundamental mechanisms of intraspecific communication in other social insect species.

## Materials and Methods

### Experiment 1: Effects of Varroa-infested honey bee brood extracts on hygienic uncapping behavior

#### Extract Collection

This experiment was conducted using multiple combs in a single VSH colony at the University of North Carolina at Greensboro. The locations of uncapped cells containing 5^th^ instar larvae were marked using a permanent marker and transparent plastic sheets held in place above the experimental cells with thumb tacks. Combs containing experimental cells were placed back into the colony, and recollected for mite introductions the following day. Only brood that had been capped were marked for experimental use. Capped cells were opened by cutting and lifting one side of the cell cap with a razor blade. Mites were collected from a donor colony using the standard sugar shake method previously described^94,95^, and were introduced randomly to approximately 50% of capped cells using a fine-tipped paintbrush. All mites were introduced to brood cells within 18 hours of capping to ensure proper initiation of mite oogenesis^96^. Cells were then resealed by gently pressing the cap against the cell wall with the side of the razor blade. The remaining capped cells served as controls; they were opened and resealed just as mite-infested cells, but did not receive a mite.

On the 4^th^ day post-capping, brood from 65 mite-infested cells and from 65 non-infested cells were collected. The presence or absence of a mite was confirmed for brood collected from each infested and non-infested brood cell, respectively. Each pool of 5 brood was soaked for 9 minutes in approximately 1.5 mL hexane each. The resulting extract was removed from the brood using a glass Pasteur pipette. Experimental (mite-infested) and control samples, each consisting of extracts collected from 13 pools of 5 brood, were combined into one extract per treatment group. The extracts were evaporated to dryness overnight at room temperature in a Fisher Hamilton SAFEAIRE^®^ hood and then reconstituted with 90 µl hexane. After 3 minutes, the resulting extract for each treatment group (65 µl after evaporation) was partitioned unequally into three vials, such that one vial received 45 µl, one 15 µl, and one 5 µl. These extracts thus corresponded to 45, 15, and 5 brood equivalents (BEq) respectively. Extracts were then evaporated overnight, as previously described, in preparation for the behavioral assay.

#### Behavioral Assay

The following day, extracts were transported on ice to the apiary, and reconstituted in 33 µl hexane each for three minutes. Each extract (30 µl after evaporation) was taken up in an airtight Hamilton syringe, and aliquoted (2 µl per cell) onto the wax caps of 15 randomly selected, previously unaltered cells containing larvae aged 4 days post-capping. This resulted in the administration of 3 BEq, 1 BEq, and 0.3 BEq of mite-infested or control brood extract. As controls, additional wax caps were either treated with hexane (2 µl per cell) or left untreated (n = 30 each). The brood comb containing the experimental cells was then placed back into the colony, and uncapping was recorded after 8 hours.

#### Statistical Analysis

Chi-square analyses were used to test for differences in uncapping between mite-infested and non-infested treatment groups for each extract concentration separately, and then for each treatment group, regardless of concentration. All tests were performed using IBM SPSS Statistics, Version 25.

### Experiment 2: Effects of Varroa, DWV, and Brood Stock on Honey Bee Brood CHCs

#### Sample Collection

This experiment was conducted over three consecutive summers (2012–2014) to analyze how CHC profiles of *Varroa*-Sensitive Hygienic (VSH), Minnesota Hygienic (HYG) and unselected control (UNS) honey bee brood might be influenced by *Varroa* mites and associated viruses. Colony hygiene level was not retested in our apiary, however before we received the selected queens, the breeders confirmed that all VSH queens exhibited the VSH trait, and all HYG queens tested above 95% removal in FKB assays. Additional data were collected for UNS brood in 2017. As with subsequent experiments, all sample collection and analysis was conducted at the University of North Carolina at Greensboro. Queens from VSH (n = 6, 2 each in 2012, 2013 and 2014), HYG (n = 8, 4 in 2012, and 2 each in 2013 and 2014), and UNS (n = 7, 4 in 2012, 2 in 2014, and 1 in 2017) stocks were caged on wax foundation combs with empty wax cells (1 queen per frame, new queens used each year). All combs were constructed in UNS colonies immediately before the experiment. Queen cages were removed from frames once eggs were laid in more than 75% of cells. In 2013, UNS queens did not lay eggs as expected, and therefore no data are available from that year for this group. After allowing 5 to 6 days for larval development, the locations of uncapped cells containing 5^th^ instar larvae were marked as described above. Frames were placed back in the colonies for cell capping.

Within 16 hours of cell capping^96^, a mite, wound, or control treatment was administered to each marked cell in each frame^97^, and the location of each cell was recorded. Mites introduced to cells were collected from adult worker bees in non-experimental colonies by sugar shake, as described above. Non-lethal wounds that mimic *Varroa* mite feeding were administered within the brood cell using 50 μm diameter needles that mimic mite-inflicted feeding sites^98^. After opening the cell capping, wounds were administered on the dorsal side of the brood between the first abdominal segment and the second thoracic segment according to existing protocols^99^. Control cells were opened just as mite and wound cells, but received neither mite nor wound treatment. All cells were sealed directly after treatment administration by pressing the cell cap against the cell wall with the edge of the razor blade.

Experimental combs were returned to their colony of origin for 24 hours to allow for complete resealing of the cell caps. Combs were then transferred to an incubator maintained at 34 °C and 50% relative humidity (RH). Each year, depending on availability, three to ten individuals from each brood stock-by-treatment group were collected each day on days 4, 5, and 6 post-capping. These days were chosen because infested brood removal rates were highest on these three days in a preliminary behavioral study (data not shown). After careful removal of the cell cap and a portion of the cell wall, each individual brood was gently removed from its cell using wide tipped, flexible forceps. Any mites on the brood were removed, and the brood was placed in a 2 mL screw top glass vial with a silicone septum (Agilent). Extraction of brood cuticular chemicals was performed within one hour of brood collection for each individual brood, and brood that were visibly damaged (e.g.: became discolored before or during chemical extraction) were excluded from the analysis. Brood labeled as receiving the mite treatment were only analyzed further when a live mite was found inside the cell at the time of collection, and brood labeled as controls were only analyzed if no mite was found in the cell at the time of collection. Total sample sizes for UNS, HYG and VSH brood were 128, 163, and 160, respectively. Mite, wound, and control sample sizes were 67, 31, and 30 for UNS brood, 54, 55, and 54 for HYG brood, and 56, 53, and 51 for VSH brood.

#### Cuticular Chemistry

Individual brood were submerged and soaked for 9 minutes in 0.5 to 0.75 mL hexane, depending on bee size. The hexane extract was removed using a glass Pasteur pipette and stored in a separate 2 mL glass vial. Brood and hexane samples were subsequently stored at −80 °C until analysis.

For extract analysis, the hexane was evaporated to dryness overnight in a fume hood and the residues were reconstituted with 100 µL (2012 and 2013) or 50 µL (2014 and 2017) of heptane after complete evaporation. Heptane used for reconstitution was spiked with butyl butyrate (1 µL butyl butyrate per 10 mL heptane) as an internal standard (IS). For reconstitution, IS-spiked heptane was added to each sample vial and left for 3 minutes, and the resulting sample was transferred into a 200 µL glass flat bottomed vial insert (Agilent). Glass inserts were used to facilitate operation of the gas Chromatography Mass Spectrometry (GC-MS) autosampler. Use of heptane rather than hexane reduced evaporation of the sample during analysis. All samples were analyzed on a Shimadzu gas chromatograph coupled to a mass spectrometer (model #QP2010S) operated at 0.97 kV, with a scan range 40 to 650 m/z. Source and interface temperatures were 230 °C and 250 °C respectively. A Zebron ZB-5MS column (30 m × 0.25 mm diameter, 0.5 μm stationary phase thickness) was used with helium as the carrier gas (column head pressure 70.2 kPa, total flow 18.1 ml/min, column flow 1.05 ml/min, linear velocity 37.8 cm/sec, purge flow 0.5 mL/min). The initial oven temperature was 80 °C, injection temperature was 280 °C and injections were made in splitless mode. After a 1 minute hold, the oven temperature was programmed from 80 to 165 °C at 15 °C/min, and then from 165 to 320 °C at 10 °C/min, with a final hold at 320 °C for 10 minutes. Compounds were identified from interpretation of their mass spectra in combination with their retention indices relative to linear alkane standards. For analyses, only the 33 cuticular chemicals that were reproducibly quantifiable in each of 10 randomly selected samples from 2012 were used for analysis because qualitative differences in chemical profiles between honey bee brood were not expected^49,70^. For quantitative analysis, the proportion of each chemical relative to the total quantity of cuticular chemicals and the IS was calculated. Arcsin transformation was performed for normalization of the proportion data and stabilization of variance^100^, according to the following equation:

$$Peak\,Proportion={\sin }^{-1}(\sqrt{\frac{individual\,peak\,area}{{\rm{\Sigma }}(peak\,areas)}})\times 100$$

#### Virus Quantification

The quantity of DWV in individual honey bee brood previously used for extract collection was analyzed. Sample sizes were 91, 126, and 124 for UNS, HYG, and VSH brood, respectively. Sample sizes were 140, 102, and 99 for control, wound, and mite treatments, respectively. For each sample, RNA was extracted, cDNA was synthesized, and quantitative PCR (qPCR) was performed. Most samples were processed with the Sensifast^TM^ cDNA Synthesis Kit and Sensifast^TM^ SYBR Hi ROX Kit. Thus, individual brood were transferred from glass vials to 2 mL Eppendorf^®^ tubes and homogenized with 0.5 mL TRIzol^TM^ (Ambion by Life Technologies) using a plastic pestle. Samples were incubated at room temperature for 10 minutes. After incubation, another 0.5 mL TRIzol^TM^ was added to each sample, and then samples were vigorously vortexed for twenty seconds. Next, 0.2 mL chloroform was added to each sample, and samples were vortexed again. Samples were incubated at room temperature for 3 minutes, and then centrifuged at 12,000 RCF for 15 minutes. The aqueous top layer of each sample was transferred into a new tube and total RNA was precipitated via centrifuge with isopropanol, washed with ethanol, dried, and resuspended in 100 µL of molecular grade water (G Biosciences).

The concentration of each RNA extract was analyzed using a Nanodrop ND-1000 Spectrophotometer and 2,000 ng of RNA were used for cDNA synthesis using the SensiFAST^TM^ cDNA Synthesis Kit (Bioline), following the manufacturer’s recommendations. The cDNA served as template in qPCR to determine the quantity of DWV in each sample. For each 2013 sample, 1 µL of cDNA, 10 µL Power SYBR Green Mix (Applied Biosystems), 8 µL of water, 0.5 µL of DWV forward primer (sequence: 5′-GAGATTGAAGCGCATGAACA-3′), and 0.5 µL of DWV reverse primer (sequence: 5′-TGAATTCAGTGTCGCCCATA-3′) were mixed in 0.1 ml MicroAmp Fast Optical 96-Well Reaction Plate tubes. For each 2014 sample, 2 µL of cDNA, 10 µL of 2x Sensifast^TM^ SYBR Hi-ROX Mix, 7.2 µL of water, 0.4 µL of forward primer, and 0.4 µL of reverse primer were mixed. All reactions were performed using 40 cycles on an Applied Biosystems StepOne Plus qPCR machine set to SYBR as the passive agent. Samples were also analyzed for the reference gene Actin (forward primer sequence: 5′-TTGTATGCCAACACTGTCCTTT-3′; reverse primer sequence: 5′-TGGCGCGATGATCTTAATTT-3′). Each reaction was run in triplicate.

Average cycle threshold (C_T_) counts were calculated for actin and DWV in each sample. When no C_T_ value was determined, a C_T_ of 40 was used. Delta C_T_ was calculated using the following equation, such that the higher the Delta C_T_ value, the greater the amount of DWV in the sample:

$$Delta\,{C}_{T}={C}_{T}(Actin)-{C}_{T}(DWV)$$

#### Statistical Analysis

General Linear Models were used to analyze the relationships between treatment (mite, wound, and control), DWV quantity, and the chemical profiles of honey bee brood from three different stocks (VSH, HYG, UNS). First, a MANOVA was performed to test treatment effect on brood chemicals across all study years, brood ages, and brood stocks. Separate one-way ANOVAs were used to evaluate the chemicals significantly affected by treatment. Bonferroni post-hoc comparisons were used to explore differences in chemical quantities between the three treatment groups (mite, wound, and control). Next, separate one-way ANOVAs were used to determine which chemicals were significantly affected by treatment for each brood stock, separately. For those chemicals significantly affected by treatment, Bonferroni post-hoc comparisons were used to explore differences in chemical proportions between the three treatment groups (mite, wound, and control). These analyses were repeated without the wound treatment to facilitate clear reporting of the direction of differences in compound quantities between mite-infested and negative control brood.

A two-way ANOVA with Bonferroni correction was used to assess the effects of brood stock and treatment on DWV. Bonferroni-corrected Pearson correlation coefficients were used to test the effect of DWV quantity on chemical quantities across all brood stocks. Pearson correlation coefficients were then used to test the effect of DWV on chemical quantities for each brood stock separately. Finally, a MANOVA was used to test for a general stock effect in control brood. This was followed by separate one-way ANOVAs with Bonferroni post-hoc comparisons to explore differences in chemical quantities between control brood in the three stocks. Bonferroni-corrected Shapiro-Wilk tests for normality were run for each year by treatment, by stock combination. P-values for the overall DWV effect are significant at the alpha 0.01 level. Bonferroni-corrected p-values were adjusted so that significance can be compared at the alpha 0.05 level. All statistical analyses were performed using IBM SPSS Statistics, Version 25.

### Experiment 3: Hygiene-associated Brood CHCs

#### Sample Collection

To relate brood CHCs directly to hygienic behavior, we compared the proportions of the previously identified CHCs between uncapped mite-infested brood, capped mite-infested brood, and capped non-infested brood in three replicate trials in separate years (2014, 2015, and 2017). As described above, mite and control treatments were applied to recently capped cells in experimental VSH colonies (two colonies in 2014 and one each in 2015 and 2017). The cells containing introduced mites were monitored every two to three hours during the day for uncapping from day 4 to day 6 post-capping. Brood was collected from mite-infested cells that were found uncapped and unharmed (i.e., nurse bees had not yet begun to remove brood). Each time an uncapped, mite-infested brood was collected, two control brood were also collected – one from a mite-infested cell that was not targeted by hygienic uncapping, and one from an equally intact control cell, without a mite. This design allowed for an age-controlled comparison of the three cell types (n = 30 each). All brood were processed for chemical extraction as described above.

As described above, individual brood were submerged in hexane for 9 minutes to extract non-polar cuticular compounds. All methodology used for chemical extraction and analysis followed the protocol described above for Experiment 2. As described above, individual brood were also analyzed for DWV content. All methodology used for RNA extraction, cDNA synthesis, RT-qPCR, and RT-qPCR analysis was as listed above.

#### Statistical Analysis

A General Linear Model was used to understand the effect of cell type (uncapped mite-infested, capped mite-infested, and capped control) on the chemical profiles of honey bee brood. First, a MANOVA was performed to test cell type effect on chemical proportions across all study years. Separate one-way ANOVAs were then used to evaluate the chemicals significantly affected by cell type. For those chemicals significantly affected by cell type, Bonferroni post-hoc comparisons were used to explore differences in proportions of chemicals between the three cell types (uncapped mite-infested, capped mite-infested, and capped control). As with the DWV analysis above, we first included all cell types, and then removed the capped control treatment to facilitate comparison of the capped and uncapped mite-infested cells. ANOVAs were performed to test the relationship between DWV quantity and cell type. All statistical analyses were performed using IBM SPSS Statistics, Version 25.

## Supplementary information

Supplementary Information

## Data Availability

The datasets generated during and/or analyzed during the current study are available from the corresponding author upon request.

## References

[1] Aizen MA, Garibaldi LA, Cunningham SA, Klein AM (2008). Long-term global trends in crop yield and production reveal no current pollination shortage but increasing pollinator dependency. Current Biology.

[2] Aizen MA, Harder LD (2009). The global stock of domesticated honey bees is growing slower than agricultural demand for pollination. Current Biology.

[3] Das, A., Sau, S., Pandit, M. K. & Saha, K. A review on: Importance of pollinators in fruit and vegetable production and their collateral jeopardy from agro-chemicals (2018).

[4] Nazzi F (2012). Synergistic parasite-pathogen interactions mediated by host immunity can drive the collapse of honeybee colonies. PLoS Pathogens.

[5] Potts SG (2010). Global pollinator declines: trends, impacts and drivers. Trends in ecology & evolution.

[6] Anderson D, Trueman J (2000). *Varroa jacobsoni* (Acari: Varroidae) is more than one species. Experimental & Applied Acarology.

[7] Rosenkranz P, Aumeier P, Ziegelmann B (2010). Biology and control of *Varroa destructor*. Journal of Invertebrate Pathology.

[8] Wilfert L (2016). Deformed wing virus is a recent global epidemic in honeybees driven by Varroa mites. Science.

[9] Ifantidis MD (1988). Some aspects of the process of *Varroa jacobsoni* mite entrance into honey bee (*Apis mellifera*) brood cells. Apidologie.

[10] Amdam GV, Hartfelder K, Norberg K, Hagen A, Omholt SW (2004). Altered physiology in worker honey bees (Hymenoptera: Apidae) infested with the mite *Varroa destructor* (Acari: Varroidae): a factor in colony loss during overwintering?. Journal of Economic Entomology.

[11] De Jong D, De Jong P, Goncalves L (1982). Weight loss and other damage to developing worker honeybees from infestation with *Varroa jacobsoni*. Journal of Apicultural Research.

[12] Garedew A, Schmolz E, Lamprecht I (2004). The energy and nutritional demand of the parasitic life of the mite *Varroa destructor*. Apidologie.

[13] Bowen-Walker P, Martin S, Gunn A (1999). The Transmission of Deformed Wing Virus between Honeybees (*Apis mellifera* L.) by the Ectoparasitic Mite *Varroa jacobsoni* Oud. Journal of Invertebrate Pathology.

[14] Chen Y, Pettis JS, Evans JD, Kramer M, Feldlaufer MF (2004). Transmission of Kashmir bee virus by the ectoparasitic mite *Varroa destructor*. Apidologie.

[15] Kanbar G, Engels W (2003). Ultrastructure and bacterial infection of wounds in honey bee (*Apis mellifera*) pupae punctured by Varroa mites. Parasitology Research.

[16] Martin SJ (2012). Global honey bee viral landscape altered by a parasitic mite. Science.

[17] Yang X, Cox-Foster D (2007). Effects of parasitization by *Varroa destructor* on survivorship and physiological traits of *Apis mellifera* in correlation with viral incidence and microbial challenge. Parasitology.

[18] Gisder S, Aumeier P, Genersch E (2009). Deformed wing virus: replication and viral load in mites (*Varroa destructor*). Journal of General Virology.

[19] Bailey, L. & Ball, B. *Honey Bee Pathology*. (Academic Press, 1991).

[20] Boecking O, Genersch E (2008). Varroosis–the ongoing crisis in bee keeping. Journal für Verbraucherschutz und Lebensmittelsicherheit.

[21] De Miranda JR, Genersch E (2010). Deformed Wing Virus. Journal of Invertebrate Pathology.

[22] Ryabov EV (2014). A virulent strain of deformed wing virus (DWV) of honeybees (*Apis mellifera*) prevails after *Varroa destructor*-mediated, or *in vitro*, transmission. PLOS Pathogens.

[23] Spivak M, Reuter GS (2001). Resistance to American foulbrood disease by honey bee colonies *Apis mellifera* bred for hygienic behavior. Apidologie.

[24] Wilson-Rich N, Spivak M, Fefferman NH, Starks PT (2009). Genetic, individual, and group facilitation of disease resistance in insect societies. Annual Review of Entomology.

[25] Rothenbuhler WC (1964). Behaviour genetics of nest cleaning in honey bees. I. Responses of four inbred lines to disease-killed brood. Animal Behaviour.

[26] Spivak M, Masterman R, Ross R, Mesce KA (2003). Hygienic behavior in the honey bee (*Apis mellifera* L.) and the modulatory role of octopamine. Journal of neurobiology.

[27] Spivak M (1996). Honey bee hygienic behavior and defense against *Varroa jacobsoni*. Apidologie.

[28] Harris JW, Danka RG, Villa JD (2010). Honey bees (Hymenoptera: Apidae) with the trait of varroa sensitive hygiene remove brood with all reproductive stages of varroa mites (Mesostigmata: Varroidae). Annals of the Entomological Society of America.

[29] Spivak M, Reuter GS (2001). *Varroa destructor* infestation in untreated honey bee (Hymenoptera: Apidae) colonies selected for hygienic behavior. Journal of Economic Entomology.

[30] Ibrahim A, Reuter GS, Spivak M (2007). Field trial of honey bee colonies bred for mechanisms of resistance against *Varroa destructor*. Apidologie.

[31] Collins AM, Pettis JS, Wilbanks R, Feldlaufer MF (2004). Performance of honey bee (*Apis mellifera*) queens reared in beeswax cells impregnated with coumaphos. Journal of Apicultural Research.

[32] Haarmann T, Spivak M, Weaver D, Weaver B, Glenn T (2002). Effects of fluvalinate and coumaphos on queen honey bees (Hymenoptera: Apidae) in two commercial queen rearing operations. Journal of Economic Entomology.

[33] Pettis J, Shimanuki WWH, Teel P (1991). Fluvalinate treatment of queen and worker honey bees (*Apis mellifera L*) and effects on subsequent mortality, queen acceptance and supersedure. Apidologie.

[34] Pettis JS, Collins AM, Wilbanks R, Feldlaufer MF (2004). Effects of coumaphos on queen rearing in the honey bee, *Apis mellifera*. Apidologie.

[35] Sylvester, H. A., Watts, R. P., De Guzman, L., Stelzer, J. A. & Rinderer, T. E. *Varroa* in the mating yard: II. The effects of Varroa and fluvalinate on drone mating competitiveness. *American Bee Journal* (1999).

[36] Sammataro D, Untalan P, Guerrero F, Finley J (2005). The resistance of varroa mites (Acari: Varroidae) to acaricides and the presence of esterase. International Journal of Acarology.

[37] Boecking O, Bienefeld K, Drescher W (2000). Heritability of the Varroa‐specific hygienic behaviour in honey bees (Hymenoptera: Apidae). Journal of Animal Breeding and Genetics.

[38] Dietemann V (2012). *Varroa destructor*: research avenues towards sustainable control. Journal of Apicultural Research.

[39] Rinderer TE, Harris JW, Hunt GJ, De Guzman LI (2010). Breeding for resistance to *Varroa destructor* in North America. Apidologie.

[40] Tsuruda JM, Harris JW, Bourgeois L, Danka RG, Hunt GJ (2012). High-resolution linkage analyses to identify genes that influence Varroa sensitive hygiene behavior in honey bees. PloS One.

[41] Gramacho KP, Spivak M (2003). Differences in olfactory sensitivity and behavioral responses among honey bees bred for hygienic behavior. Behavioral Ecology and Sociobiology.

[42] Masterman R, Ross R, Mesce K, Spivak M (2001). Olfactory and behavioral response thresholds to odors of diseased brood differ between hygienic and non-hygienic honey bees (*Apis mellifera* L.). Journal of Comparative Physiology A.

[43] Goode K, Huber Z, Mesce KA, Spivak M (2006). Hygienic behavior of the honey bee (*Apis mellifera*) is independent of sucrose responsiveness and foraging ontogeny. Hormones and Behavior.

[44] Hu H (2016). Proteome analysis of the hemolymph, mushroom body, and antenna provides novel insight into honeybee resistance against Varroa infestation. Journal of proteome research.

[45] Mondet F (2015). Antennae hold a key to *Varroa*-sensitive hygiene behaviour in honey bees. Scientific Reports.

[46] Gempe T, Stach S, Bienefeld K, Otte M, Beye M (2016). Behavioral and molecular studies of quantitative differences in hygienic behavior in honeybees. BMC Research Notes.

[47] Aumeier P, Rosenkranz P (2001). Scent or movement of *Varroa destructor* mites does not elicit hygienic behaviour by Africanized and Carniolan honey bees. Apidologie.

[48] Richard F, Aubert A, Grozinger C (2008). Modulation of social interactions by immune stimulation in honey bee, *Apis mellifera*, workers. BMC Biology.

[49] Salvy M (2001). Modifications of the cuticular hydrocarbon profile of *Apis mellifera* worker bees in the presence of the ectoparasitic mite *Varroa jacobsoni* in brood cells. Parasitology.

[50] Schöning C (2012). Evidence for damage-dependent hygienic behaviour towards *Varroa destructor*-parasitised brood in the western honey bee, *Apis mellifera*. The Journal of Experimental Biology.

[51] Harbo JR, Harris JW (2009). Responses to Varroa by honey bees with different levels of Varroa Sensitive Hygiene. Journal of Apicultural Research.

[52] Mondet F (2016). Specific cues associated with honey bee social defence against Varroa destructor infested brood. Scientific reports.

[53] Le Conte Y (2015). *Varroa destructor* changes its cuticular hydrocarbons to mimic new hosts. Biology Letters.

[54] Martin C (2001). Variations in chemical mimicry by the ectoparasitic mite *Varroa jacobsoni* according to the developmental stage of the host honey-bee *Apis mellifera*. Insect Biochemistry and Molecular Biology.

[55] Nation J, Sanford M, Milne K (1992). Cuticular hydrocarbons from *Varroa jacobsoni*. Experimental & Applied Acarology.

[56] Gilliam M, Taber S, Richardson GV (1983). Hygienic behavior of honey bees in relation to chalkbrood disease. Apidologie.

[57] Gilliam M, Taber S, Lorenz BJ, Prest DB (1988). Factors affecting development of chalkbrood disease in colonies of honey bees, *Apis mellifera*, fed pollen contaminated with *Ascosphaera apis*. Journal of Invertebrate Pathology.

[58] Wagoner, K. M., Spivak, M. & Rueppell, O. Brood Affects Hygienic Behavior in the Honey Bee (Hymenoptera: Apidae). *Journal of economic entomology* (2018). 10.1093/jee/toy266 30212863

[59] McAfee A (2018). A death pheromone, oleic acid, triggers hygienic behavior in honey bees (Apis mellifera L.). Scientific Reports.

[60] He XJ (2016). Starving honey bee (Apis mellifera) larvae signal pheromonally to worker bees. Scientific reports.

[61] Wilson EO, Durlach NI, Roth LM (1958). Chemical releasers of necrophoric behavior in ants. Psyche.

[62] Rollo C, Czvzewska E, Borden J (1994). Fatty acid necromones for cockroaches. Naturwissenschaften.

[63] Annoscia D, Del Piccolo F, Nazzi F (2012). How does the mite *Varroa destructor* kill the honeybee *Apis mellifera*? Alteration of cuticular hydrcarbons and water loss in infested honeybees. Journal of Insect Physiology.

[64] Blomquist, G. J. & Bagnères, A.-G. *Insect hydrocarbons: biology, biochemistry, and chemical ecology*. (Cambridge University Press, 2010).

[65] Ferreira-Caliman M, Turatti I, Lopes N, Zucchi R, Nascimento F (2012). Analysis of insect cuticular compounds by non-lethal solid phase micro extraction with styrene-divinylbenzene copolymers. Journal of Chemical Ecology.

[66] Soroker V, Hefetz A (2000). Hydrocarbon site of synthesis and circulation in the desert ant *Cataglyphis niger*. Journal of Insect Physiology.

[67] Howard, R. W. *Cuticular hydrocarbons and chemical communication*. (University of Nebraska Press, 1993).

[68] Howard RW, Blomquist GJ (2005). Ecological, behavioral, and biochemical aspects of insect hydrocarbons. Annu. Rev. Entomol..

[69] Lockey KH (1988). Lipids of the insect cuticle: origin, composition and function. Comparative Biochemistry and Physiology Part B: Comparative Biochemistry.

[70] Hefetz A (2007). The evolution of hydrocarbon pheromone parsimony in ants (Hymenoptera: Formicidae)—interplay of colony odor uniformity and odor idiosyncrasy. Myrmecological News.

[71] Dani FR, Jones GR, Destri S, Spencer SH, Turillazzi S (2001). Deciphering the recognition signature within the cuticular chemical profile of paper wasps. Animal Behaviour.

[72] LeConte Y, Hefetz A (2008). Primer pheromones in social hymenoptera. Annu. Rev. Entomol..

[73] Nascimento D, Nascimento F (2012). Acceptance threshold hypothesis is supported by chemical similarity of cuticular hydrocarbons in a stingless bee, *Melipona asilvai*. Journal of Chemical Ecology.

[74] Baracchi D, Fadda A, Turillazzi S (2012). Evidence for antiseptic behaviour towards sick adult bees in honey bee colonies. Journal of Insect Physiology.

[75] Nazzi F, Della Vedova G, D’Agaro M (2004). A semiochemical from brood cells infested by Varroa destructor triggers hygienic behaviour in Apis mellifera. Apidologie.

[76] Gibbs AG (1998). Water-proofing properties of cuticular lipids. American Zoologist.

[77] Martin C (2002). Potential mechanism for detection by *Apis mellifera* of the parasitic mite *Varroa destructor* inside sealed brood cells. Physiological Entomology.

[78] Larsson K, Norén B, Odham G (1975). Antimicrobial effect of simple lipids with different branches at the methyl end group. Antimicrobial agents and chemotherapy.

[79] Villa JD, Danka RG, Harris JW (2009). Simplified methods of evaluating colonies for levels of Varroa Sensitive Hygiene (VSH). Journal of Apicultural Research.

[80] Kevill JL, Highfield A, Mordecai GJ, Martin SJ, Schroeder DC (2017). ABC assay: method development and application to quantify the role of three DWV master variants in overwinter colony losses of European honey bees. Viruses.

[81] Lipsitch M, Siller S, Nowak MA (1996). The evolution of virulence in pathogens with vertical and horizontal transmission. Evolution.

[82] Clarke DK (1994). The red queen reigns in the kingdom of RNA. viruses. Proceedings of the National Academy of Sciences.

[83] Spötter A, Gupta P, Nürnberg G, Reinsch N, Bienefeld K (2012). Development of a 44K SNP assay focussing on the analysis of a varroa‐specific defence behaviour in honey bees (*Apis mellifera* carnica). Molecular Ecology Resources.

[84] Spivak M, Downey DL (1998). Field assays for hygienic behavior in honey bees (Hymenoptera: Apidae). Journal of Economic Entomology.

[85] Danka RG, Harris JW, Villa JD, Dodds GE (2013). Varying congruence of hygienic responses to *Varroa destructor* and freeze-killed brood among different types of honeybees. Apidologie.

[86] Pull, C. D. *et al*. Destructive Disinfection Of Infected Brood Prevents Systemic Disease Spread In Ant Colonies. *bioRxiv*, 116657 (2017). 10.7554/eLife.32073 PMC5760203 29310753

[87] Doublet V (2017). Unity in defence: honeybee workers exhibit conserved molecular responses to diverse pathogens. BMC genomics.

[88] Langstroth, L. L. *Langstroth on the Hive and the Honey-bee: A Bee-keeper’s Manual*. (Hopkins, Bridgman & Company, 1914).

[89] Kirrane MJ (2011). Asynchronous development of honey bee host and *Varroa destructor* (Mesostigmata: Varroidae) influences reproductive potential of mites. Journal of Economic Entomology.

[90] Harris JW, Danka RG, Villa JD (2012). Changes in infestation, cell cap condition, and reproductive status of *Varroa destructor* (Mesostigmata: Varroidae) in brood exposed to honey bees with Varroa sensitive hygiene. Annals of the Entomological Society of America.

[91] Boecking O, Drescher W (1992). The removal response of *Apis mellifera* L. colonies to brood in wax and plastic cells after artificial and natural infestation with *Varroa jacobsoni* Oud. and to freeze-killed brood. Experimental & Applied Acarology.

[92] Genersch E (2010). The German bee monitoring project: a long term study to understand periodically high winter losses of honey bee colonies. Apidologie.

[93] Harbo JR (1986). Effect of population size on brood production, worker survival and honey gain in colonies of honeybees. Journal of Apicultural Research.

[94] Fakhimzadeh K (2001). Effectiveness of confectioner sugar dusting to knock down Varroa destructor from adult honey bees in laboratory trials. Apidologie.

[95] Dietemann V (2013). Standard methods for varroa research. Journal of apicultural research.

[96] Frey E, Odemer R, Blum T, Rosenkranz P (2013). Activation and interruption of the reproduction of Varroa destructor is triggered by host signals (Apis mellifera). Journal of invertebrate pathology.

[97] Kuster RD, Boncristiani HF, Rueppell O (2014). Immunogene and viral transcript dynamics during parasitic *Varroa destructor* mite infection of developing honey bee (*Apis mellifera*) pupae. The Journal of Experimental Biology.

[98] Herrmann M, Kanbar G, Engels W (2005). Survival of honey bee (*Apis mellifera*) pupae after trypan blue staining of wounds caused by *Varroa destructor* mites or artificial perforation. Apidologie.

[99] Dade, H. A. *Anatomy and dissection of the honey bee* Revised Edition edn, (International Bee Research Association, 2009).

[100] Sokal, R. R. & Rohlf, F. J. *The principles and practice of statistics in biological research*. (WH Freeman and Co., 1995).

