# Supplementary material for: Stock-specific chemical brood signals are induced by Varroa and Deformed Wing Virus, and elicit hygienic response in the honey bee
Source: Sci Rep. 2019 Jun 19;9:8753. doi: 10.1038/s41598-019-45008-2 (PMC6584651; doi:10.1038/s41598-019-45008-2)
Supplement: Supplementary file 1 — Supplementary Information [file 41598_2019_45008_MOESM1_ESM.pdf]

Stock-specific chemical brood signals are induced by *Varroa* and Deformed Wing Virus, and elicit hygienic response in the honey bee.

Wagoner K.*[1], Spivak M.[2], Hefetz A.[3], Reams T.[4], Rueppell O.[1]

[1]Biology Department, University of North Carolina at Greensboro

[2]Department of Entomology, University of Minnesota

[3]George S. Wise Faculty of Life Science, Tel Aviv University

[4]Department of Entomology, Texas A&M University

Supplementary Data

Supp. Table S1.

Summary of statistics associated with CHC analyses from Experiments II and III. Statistical data for the overall effects on relative CHC abundance of treatment (mite, wound, and control) and DWV over all brood types are provided. Also provided are the statistical data for effects on relative CHC abundance of mite (mite vs. control), DWV, and hygienic behavior (uncapped mite vs. capped mite) for each brood type separately. Yellow and blue cells indicate significant increases and decreases in relative CHC abundance, respectively.

	Overall Treatment Effect		Overall DWV Effect		Experiment 2 Mite Effect						Experiment 2 DWV Effect						Experiment 3 Hygiene Effect	
					UNS		HYG		VSH		UNS		HYG		VSH		VSH	
PEAK	$F_{(2,409)}$	p	Pearson's R	p	$F_{(1,57)}$	p	$F_{(1,106)}$	p	$F_{(1,104)}$	p	Pearson's R	p	Pearson's R	p	Pearson's R	p	$F_{(1,58)}$	p
unidentified	1.064	0.346	0.263	<0.001	0.105	0.747	0.608	0.437	0.214	0.644	0.061	0.665	0.302	0.001	0.237	0.008	0.559	0.458
nonadecane	1.734	0.178	0.115	0.034	0.194	0.662	1.791	0.184	1.623	0.205	-0.124	0.241	0.095	0.292	0.157	0.083	0.057	0.813
heneicosane	0.804	0.448	0.010	0.858	1.624	0.208	0.001	0.971	0.919	0.340	-0.036	0.733	0.063	0.485	-0.036	0.695	0.660	0.420
tricosane	0.147	0.863	0.096	0.076	0.001	0.973	0.797	0.374	0.417	0.520	0.089	0.401	0.138	0.122	0.058	0.520	0.006	0.938
9- + 11-methyltricosane	0.328	0.721	0.104	0.055	0.799	0.375	0.042	0.838	0.893	0.347	0.286	0.006	0.215	0.015	-0.002	0.986	0.044	0.834
4-methyltetracosane	0.081	0.922	0.083	0.124	0.009	0.924	0.072	0.789	0.579	0.448	0.323	0.002	0.061	0.496	0.016	0.856	0.497	0.484
pentacosene	0.505	0.604	0.208	<0.001	0.046	0.832	0.003	0.954	0.027	0.870	0.376	<0.001	0.240	0.007	0.140	0.122	1.020	0.317
pentacosane	0.539	0.584	0.188	<0.001	0.060	0.807	0.011	0.915	0.211	0.647	0.267	0.010	0.159	0.074	0.112	0.216	0.669	0.417
11- + 13-methylpentacosane	0.217	0.805	0.095	0.079	0.659	0.420	0.174	0.677	0.001	0.976	0.261	0.012	0.228	0.010	-0.048	0.596	0.647	0.424
hexacosane	0.365	0.694	0.141	0.009	0.011	0.917	0.034	0.855	0.220	0.640	0.276	0.008	0.133	0.136	0.033	0.718	0.172	0.680
12- + 14-methylhexacosane	0.203	0.816	0.092	0.091	0.718	0.400	0.085	0.771	0.005	0.943	0.238	0.023	0.208	0.019	-0.069	0.443	0.361	0.550
heptacosene	2.871	0.058	0.193	<0.001	1.286	0.262	1.373	0.244	0.879	0.351	0.286	0.006	0.309	<0.001	-0.032	0.725	1.720	0.195
heptacosane	0.678	0.508	-0.144	0.008	0.223	0.639	0.019	0.890	0.044	0.835	-0.028	0.795	-0.282	0.001	-0.119	0.188	2.947	0.091
11- + 13-methylheptacosane	2.529	0.081	-0.108	0.047	3.908	0.053	2.153	0.145	1.185	0.279	0.003	0.980	-0.021	0.818	-0.149	0.099	1.542	0.219
5-methylheptacosane	3.336	0.037	-0.088	0.104	3.374	0.071	1.847	0.177	1.378	0.243	-0.082	0.439	0.018	0.844	-0.177	0.049	0.420	0.519
11,15-dimethylheptacosane	1.676	0.188	-0.038	0.482	2.695	0.106	0.196	0.659	1.442	0.233	0.004	0.967	0.079	0.379	-0.068	0.455	0.187	0.667
7,x-dimethylheptacosane	1.605	0.202	-0.058	0.287	1.615	0.209	0.202	0.654	0.986	0.323	-0.069	0.518	-0.017	0.853	-0.039	0.664	1.447	0.234
5,x-dimethylheptacosane	1.972	0.141	-0.089	0.100	0.189	0.665	0.204	0.652	1.379	0.243	-0.102	0.335	-0.037	0.678	-0.101	0.265	0.483	0.490
octacosane	0.672	0.511	-0.026	0.638	0.922	0.341	0.113	0.738	0.054	0.816	0.017	0.872	-0.048	0.594	-0.055	0.547	0.033	0.857
nonacosene	2.492	0.084	0.041	0.447	1.938	0.169	1.942	0.166	0.861	0.356	0.116	0.272	0.117	0.192	-0.106	0.241	2.035	0.159
nonacosane	0.407	0.666	-0.157	0.004	1.055	0.309	0.057	0.812	0.080	0.777	-0.062	0.558	-0.285	0.001	-0.096	0.288	0.007	0.931
11- + 13- +15-methylnonacosane	0.699	0.498	-0.109	0.045	0.002	0.964	0.162	0.688	1.947	0.166	-0.211	0.045	-0.099	0.269	-0.016	0.864	0.109	0.743
11,17-dimethylnonacosane	1.138	0.322	0.004	0.941	0.088	0.768	0.689	0.408	1.144	0.287	0.161	0.127	-0.121	0.178	0.058	0.523	0.014	0.906
triacontene	1.165	0.313	-0.052	0.339	1.685	0.199	0.328	0.568	1.470	0.228	0.038	0.719	-0.157	0.079	0.024	0.795	0.255	0.616
triacontane	1.946	0.144	0.010	0.855	0.930	0.339	2.286	0.134	0.591	0.444	-0.088	0.409	0.040	0.656	0.021	0.816	0.012	0.915
hentriacontene	1.460	0.234	0.017	0.749	0.006	0.939	0.215	0.644	0.278	0.599	0.227	0.031	0.102	0.253	-0.138	0.127	7.375	0.009
hentriacontane	0.804	0.448	-0.043	0.429	1.256	0.267	0.108	0.743	0.780	0.379	-0.015	0.886	-0.138	0.124	-0.023	0.798	0.450	0.505
11- + 13- +15-methylhentriacontane	1.131	0.324	-0.005	0.934	0.426	0.517	0.836	0.363	0.834	0.363	-0.117	0.268	-0.002	0.982	0.032	0.728	0.098	0.755
13,17-dimethylhentriacontane	0.834	0.435	-0.064	0.240	1.400	0.242	0.532	0.468	0.261	0.610	-0.007	0.951	-0.136	0.129	0.001	0.990	0.000	0.986
dotriacontene	0.787	0.456	-0.089	0.101	0.044	0.835	0.630	0.429	0.141	0.708	0.043	0.686	-0.090	0.318	-0.164	0.069	1.877	0.176
methyldotriacontane	1.731	0.178	-0.033	0.545	0.001	0.979	0.369	0.545	1.790	0.184	-0.012	0.911	-0.088	0.326	0.015	0.871	2.867	0.096
tritriacontene	7.380	0.001	0.164	0.002	1.335	0.253	1.783	0.185	5.983	0.016	0.263	0.012	0.347	<0.001	-0.040	0.659	10.432	0.002
tritriacontane	0.917	0.401	0.063	0.247	0.381	0.540	0.007	0.933	0.401	0.528	-0.351	0.001	0.137	0.127	0.130	0.150	0.962	0.331

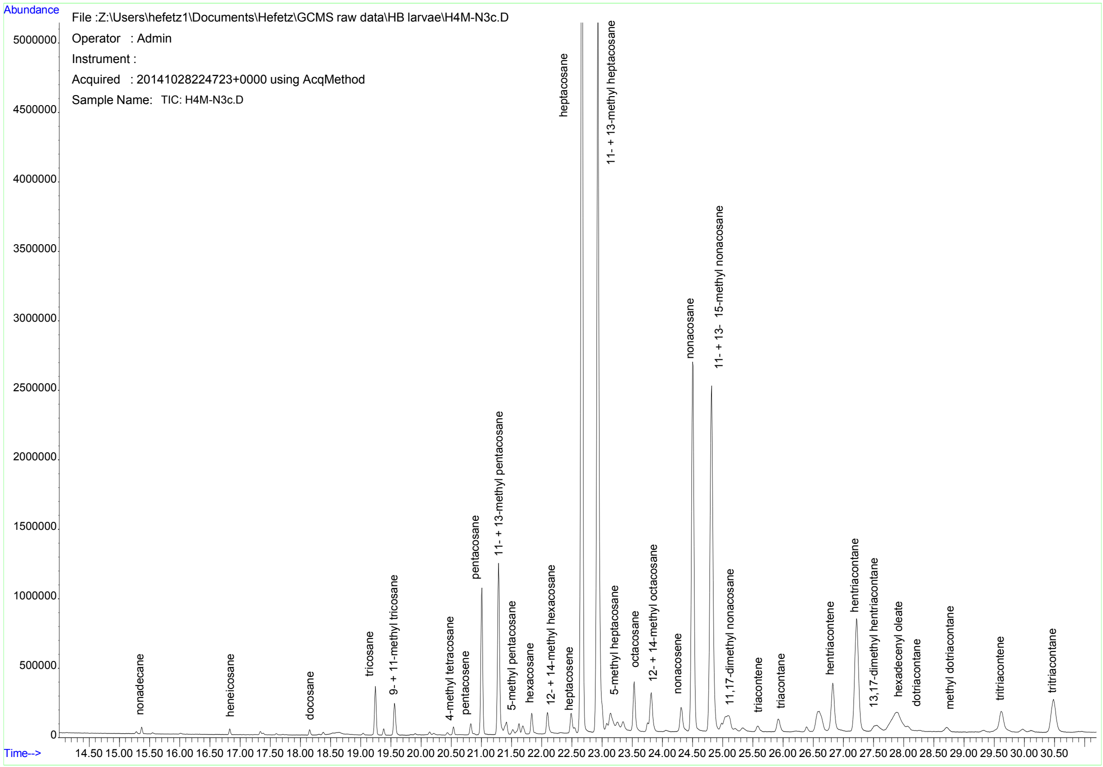

Supp. Figure S1. Annotated GC/MS spectra from a mite-infested brood sample. Retention times are shown in minutes on the x-axis, and chemical abundance is shown on the y-axis.

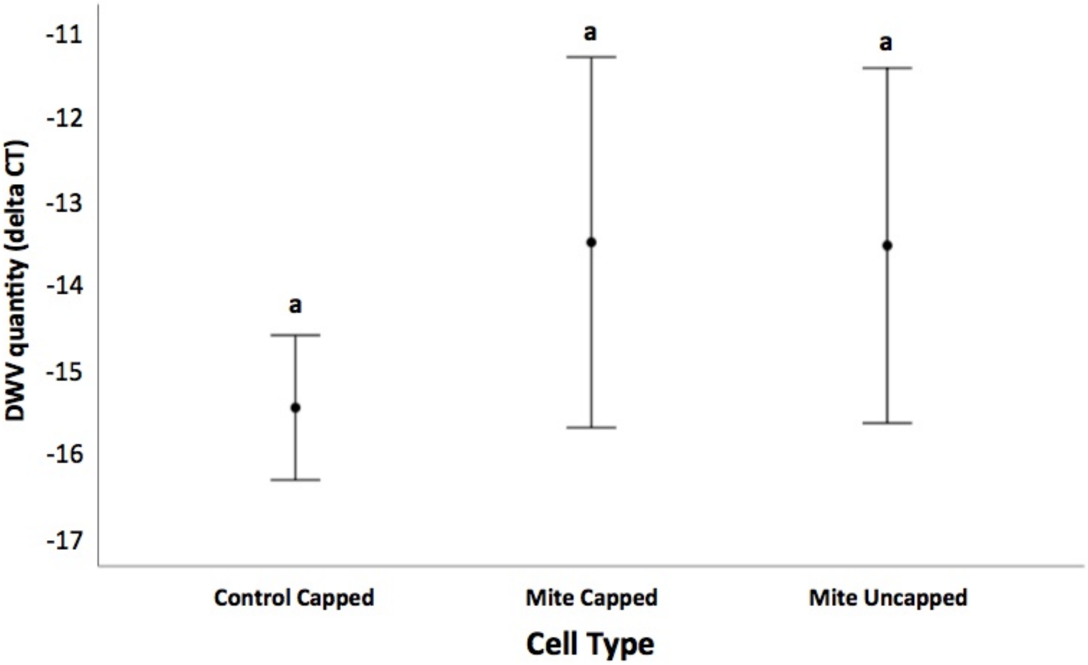

Supp. Figure S2. Mean DWV Quantity of VSH Brood from Control Capped, Mite Capped, and Mite Uncapped Cells. For each mean, 95% CI intervals are provided. No significant differences were observed between cell types, from an ANOVA with Bonferroni correction
